# Suicidal crisis in the adolescent: About 3 clinical cases

**DOI:** 10.1192/j.eurpsy.2023.1520

**Published:** 2023-07-19

**Authors:** I. Belabbes, I. Katir, M. Chtibi, H. Kisra

**Affiliations:** Arrazi hospital, Sale, Morocco

## Abstract

**Introduction:**

Suicide is the third most common cause of death among adolescents. It is linked to several mental pathologies. Early detection and effective and rapid management are essential elements to improve the mental health of the adolescent and prevent suicidal behaviour.

**Objectives:**

Our objective is :

- To discuss through these medical observations the risk factors and pathologies at high risk of suicide,
- To detail the urgent course of action to be taken in the event of suicide attempts and
- To underline the importance of early management to prevent suicide in adolescents

**Methods:**

We report hereafter the clinical cases of 3 adolescents followed in child psychiatry for the management of a suicidal crisis.

**Results:**

1st clinical case :

This is a 13 year old girl, brought back by the staff of the Lalla Meryem child protection centre, where she has been living since her separation from her family following abuse by her mother. She reports a reliving of scenes where her mother burned her private parts. She reports disturbed sleep with night terrors and nightmares. In addition, the centre’s staff reported irritability, crying spells and verbalized suicidal threats.

2nd clinical case:

This is a 15 year old girl referred by the paediatric service for management of a suicide attempt by ingestion of rat poison. She stopped her schooling 2 years ago to take care of her mother who had cervical cancer and died 6 months ago. Since then, she has experienced sadness of mood, low self-esteem with ideas of devaluation. During the first psychiatric interview, she did not criticize her suicidal act and said that she wanted to kill herself, which she considered the only solution to her suffering.

3rd clinical case:

This is a 12-year-old adolescent, followed in child psychiatry for conduct disorder with problematic use of tobacco, cannabis and benzodiazepines. He had stopped going to school since the third grade.

He consulted for a suicide attempt by ingesting 30 antihypertensive tablets. After somatic care, he was referred to us from the medical emergency department for psychiatric care.

**Conclusions:**

Suicide among adolescents is on the increase in Morocco. It is necessary to take into account the risk of suicide in the face of any mental or somatic pathology. A rapid and urgent course of action is necessary to avoid recurrence.

**Disclosure of Interest:**

None Declared

